# Supplementary figures and images for: Dietary impact on the gut resistome: western diet independently increases the prevalence of antibiotic resistance genes within the gut microbiota
Source: Microbiol Spectr. 2025 Jul 28;13(9):e02766-24. doi: 10.1128/spectrum.02766-24 (PMC12403810; doi:10.1128/spectrum.02766-24)

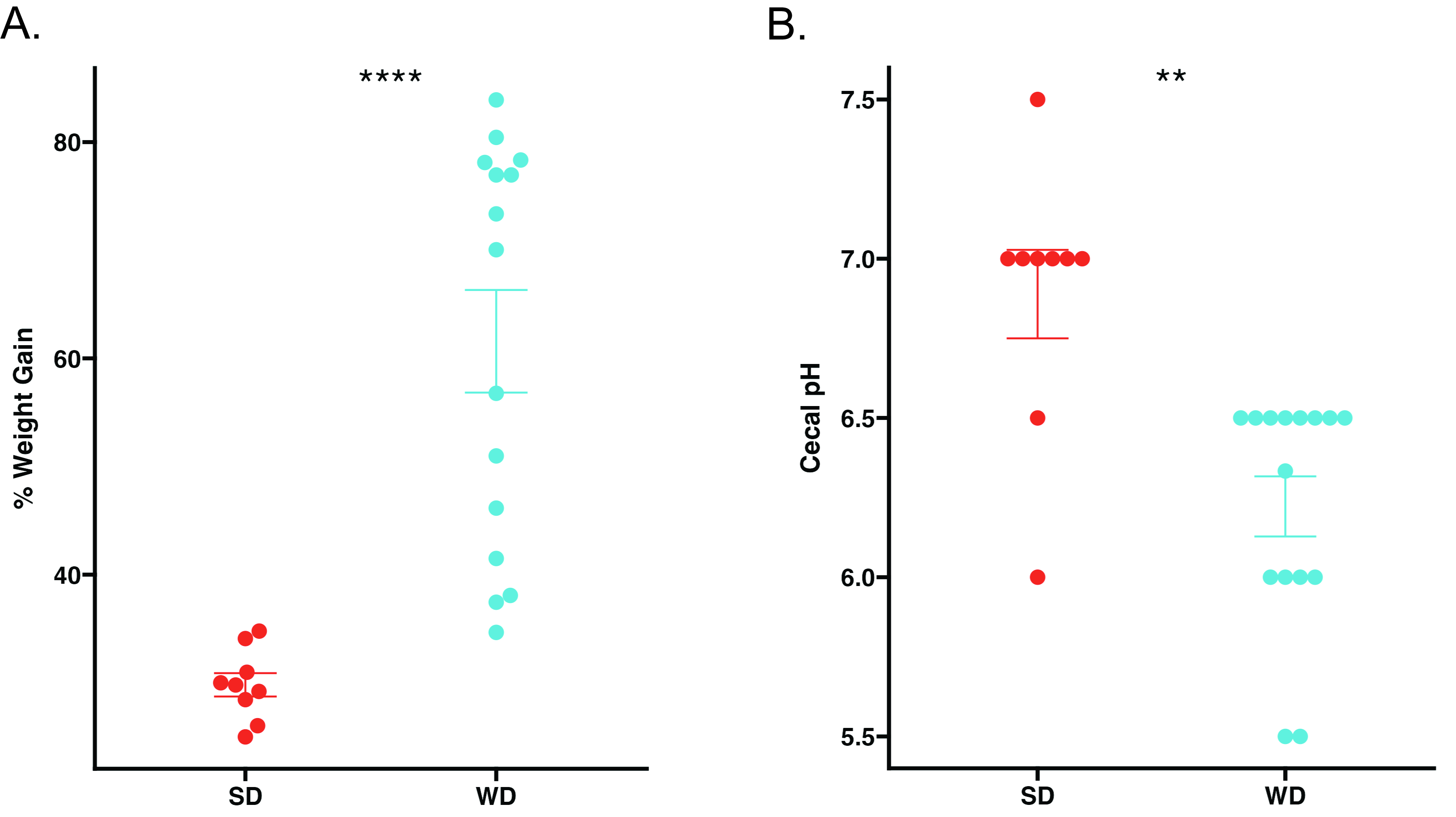

Supplement: Fig. 1 — Western diet increases weight gain and alters cecal pH compared to standard diet. [file spectrum.02766-24-s0001.tif]

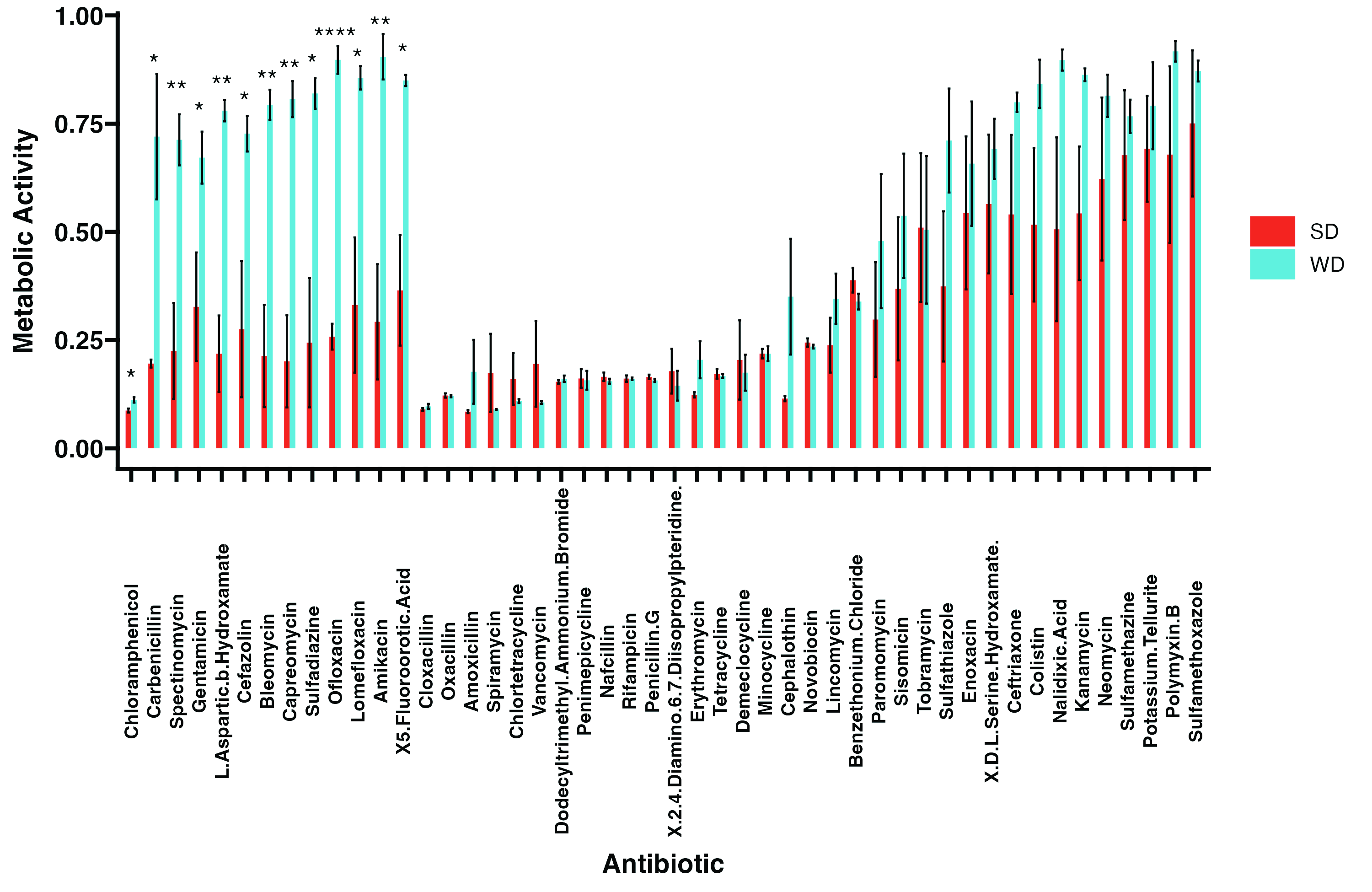

Supplement: Fig. 2 — Western diet mice stool microbiota has increased metabolic activity in the presence of antibiotics compared to SD mice stool microbiota. [file spectrum.02766-24-s0002.tif]
