# Supplementary material for: Dietary impact on the gut resistome: western diet independently increases the prevalence of antibiotic resistance genes within the gut microbiota
Source: Microbiol Spectr. 2025 Jul 28;13(9):e02766-24. doi: 10.1128/spectrum.02766-24 (PMC12403810; doi:10.1128/spectrum.02766-24)
Supplement: Supplemental material — Supplemental figure and table legends. [file spectrum.02766-24-s0003.docx]

**Supplemental Figure 1: Western diet (WD) increases weight gain and alters cecal pH compared to Standard Diet (SD)**. Western diet mice gained significantly more weight compared to SD fed mice after 7 weeks (A). Western diet significantly reduced cecal pH (B). WD n = 15, SD n = 9, ***p < 0.001, ** p < 0.01

**Supplemental Figure 2: Western diet mice stool microbiota has increased metabolic activity in the presence of antibiotics compared to SD mice stool microbiota.** The metabolic activity of stool microbiota from WD fed mice and SD mice was compared on Biolog under aerobic conditions. WD n = 5, SD n = 5, *p <0.05, ** p< 0.01, **** p <0.0001

**Supplemental Table 1:**  Chart of how antibiotics utilized on the biolog plates were grouped by antibiotic class

**Supplemental Table 2:** Sample classification for metagenomics
